# Audio in VR: Effects of a Soundscape and Movement-Triggered Step Sounds on Presence

**DOI:** 10.3389/frobt.2020.00020

**Published:** 2020-02-21

**Authors:** Angelika C. Kern, Wolfgang Ellermeier

**Affiliations:** Technical University of Darmstadt, Institute of Psychology, Darmstadt, Germany

**Keywords:** virtual reality, presence, audio, soundscape, step sounds

## Abstract

For effective virtual realities, “presence,” the feeling of “being there” in a virtual environment (VR), is deemed an essential prerequisite. Several studies have assessed the effect of the (non-)availability of auditory stimulation on presence, but due to differences in study design (e.g., virtual realities used, types of sounds included, rendering technologies employed), generalizing the results and estimating the effect of the auditory component is difficult. In two experiments, the influence of an ambient nature soundscape and movement-triggered step sounds were investigated regarding their effects on presence. In each experiment, approximately forty participants walked on a treadmill, thereby strolling through a virtual park environment reproduced via a stereoscopic head-mounted display (HMD), while the acoustical environment was delivered via noise-canceling headphones. In Experiment 1, conditions with the ambient soundscape and the step sounds either present or absent were combined in a 2 × 2 within-subjects design, supplemented with an additional “no-headphones” control condition. For the synchronous playback of step sounds, the probability of a step being taken was estimated by an algorithm using the HMD's sensor data. The results of Experiment 1 show that questionnaire-based measures of presence and realism were influenced by the soundscape but not by the reproduction of steps, which might be confounded with the fact that the perceived synchronicity of the sensor-triggered step sounds was rated rather low. Therefore, in Experiment 2, the step-reproduction algorithm was improved and judged to be more synchronous by participants. Consequently, large and statistically significant effects of both kinds of audio manipulations on perceived presence and realism were observed, with the effect of the soundscape being larger than that of including footstep sounds, possibly due to the remaining imperfections in the reproduction of steps. Including an appropriate soundscape or self-triggered footsteps had differential effects on subscales of presence, in that both affected overall presence and realism, while involvement was improved and distraction reduced by the ambient soundscape only.

## Introduction

This study evaluates the effect of auditory stimuli and their influence on presence, the feeling of “being there” in the virtual environment, while the user can actually experience walking (by using a treadmill) in the virtual world. The effect of the (non-)availability of auditory stimuli on presence has already been assessed by several studies (Hendrix and Barfield, 1996; Snow and Williges, 1998; Dinh et al., 1999; Larsson et al., 2007), but comparing and generalizing the outcomes is problematic: Some of the work dates back to some 20 years ago, which raises the question whether studies conducted with virtual realities back then can really be generalized to the more sophisticated technology used today. Also, the studies vary with respect to their visual presentation of the virtual realities [e.g., head-mounted displays (HMD's) vs. large-scale projections], the kind of sounds reproduced (e.g., background music or environmental sounds), whether the sounds were adjusted to the body- and/or head-position of the virtual agent, as well as the tools used to measure “presence.” Therefore, further studies controlling these aspects are needed for adding detail to the emerging picture. Remarkably, none of the studies reviewed was conducted while the user was literally walking—and receiving genuine proprioceptive feedback—while moving through the VR. Thus, a study assessing the influence of sound on perceived presence while actually walking appeared to be called for, thereby putting existing results into a new perspective.

For effective Virtual Realities, “presence” is deemed an essential prerequisite. There are several definitions of “presence” but in general, it can be defined as the sensation of “being there” in the mediated world. When presence is related with virtual realities, it is defined as “the perceptual illusion of non-mediation” (Lombard and Ditton, 1997). Therefore, absolute presence in VR would mean that the user is under the impression that he or she is actually being in the virtual environment no longer realizing the mediating technology exists, thus acting as if there was no medium.

Often, the term “immersion” is used interchangeably with “presence,” but we follow the definition of Slater and Wilbur (1997) that immersion is rather an objective assessment of the “characteristics of a technology,” having dimensions such as the extent to which a display system can deliver “an inclusive, extensive, surrounding, and vivid illusion of virtual environment to a participant.” By that definition, immersion describes the technical potential of presenting the virtual environment while masking the surroundings, whereas presence describes the subjective (psychological) feeling of “being there” in the environment.

Why is it important to increase the sense of presence in virtual realities? Slater and Wilbur (1997, p. 8) argue, that “the greater the degree of presence, the greater the chance that participants will behave in a VE in a manner similar to their behavior in similar circumstances in everyday reality.” For many applications (e.g., training fire-fighters or surgeons) it is crucial for the effectiveness that the user behaves similar to everyday circumstances, or, more precisely, will behave in everyday circumstances as he or she did in the virtual environment during training. In short, Cummings and Bailenson (2016, p. 3) sum up the relation of immersion and presence: “the more immersive the system, the more likely an individual will feel present within the mediated environment and the more likely that the virtual setting will dominate over physical reality in determining user responses.”

Knowing increased presence is desirable, it is eminent to know how to achieve it. Generally, the visual input is deemed to be dominating the other senses, but still, auditory perception can compensate for some constraints of purely visual perception: Larsson et al. (2010) argue that the auditory system might be less accurate than the visual one in terms of spatial resolution but in return provides spatial cues from the entire surrounding space at the same time, thereby compensating for the restricted visual field of view. Furthermore, Larsson et al. (2010) imply that the “temporal nature of sound” leads to the impression that “something is happening”—even if the visual scene may be completely static. Therefore, auditory input can complement visual input quite well, which makes it an adequate Research Topic.

To gain insight into the relevance sound actually has on perception, one might study the work of Ramsdell (1978) who interviewed World War II veterans which had experienced sudden profound hearing loss. In contrast to people whose hearing had been impaired since birth, people with sudden hearing loss have to cope with abruptly losing one of the senses they (subconsciously) depended on which allows them to describe their changed perception.

In order to structure the phenomena, Ramsdell (1978) postulates three levels at which hearing occurs: the social level, the signal/warning level and the primitive level. At the social level, hearing is used to comprehend language and abstract meaning in order to communicate and interact with others (e.g., the word “bee”). At the signal/ warning level, hearing is used as a direct sign or signal of (potentially harmful) events which allows us to keep track of what's happening and act accordingly (e.g., the buzzing sound of a bee close to us makes us jump). At the primitive level, sound acts as “the auditory background of all daily living” (e.g., a faint buzzing of bees when strolling through a garden). According to Ramsdell (1978, p. 501), the importance of the primitive level is often underrated: “These incidental noises maintain our feeling of being part of a living world and contribute to our own sense of being alive.”

The interviews lead to the conclusion that the interviewed were very much aware of having lost the first and second level of hearing, but the loss of the third, primitive level was not that obvious to them. Losing their ability to hear they reported feelings of depression, isolation and “deadness.” The question is: Does being immersed in a virtual reality without sound make us experience (some lighter form of) these feelings? Or is exposing ourselves to a virtual reality rather deliberately handing over our senses, knowing it will be short-term and even the absence of sound might be acceptable?

In the following, we will briefly review the few studies that have assessed the influence of sounds being reproduced in contrast to silence in their influence on presence in virtual realities.

Hendrix and Barfield (1996) visually presented a stereoscopic virtual room scenario by using a projector, a screen and LCD shutter glasses. The 16 participants were asked to navigate freely in the virtual environment until they felt it was familiar. The sound effects spatially reproduced via headphones were a radio, throwing coins into a vending machine and receiving a can of soda. Presence was measured by two one-item questions (scaled 1–100 and 1–5), as was perceived realism (scaled 1–5). Table 1 shows the presence ratings obtained, the group means differed significantly between conditions. For the condition where no sound was presented, realism-ratings from 1 to 5 averaged at *M* = 3.45 (*SD* = 0.82) compared to *M* = 2.73 (*SD* = 0.90) in the condition with sound. Curiously thus, presence [1–5] and realism decreased when sound was added.

**Table 1:** Effect of sound on presence-Ratings (earlier studies).

	**Presence [0–100]**			**Presence [1–5]**	**Presence [1–7]**
	**Hendrix and Barfield, 1996**	**Dinh et al., 1999**	**Larsson et al., 2007**	**Hendrix and Barfield, 1996**	**Larsson et al., 2007**
No Sound	45.45 (19.42)	63.4 (18.6)	49.15 (3.99)	3.45 (0.82)	4.45 (0.21)
Sound	56.09 (21.00)	69.3 (16.1)	57.57 (4.34)	2.73 (0.90)	5.21 (0.26)

Dinh et al. (1999) visually presented virtual office-interior scenarios using a stereoscopic HMD. In some experimental conditions, they also reproduced stereophonic sounds via headphones that fit the objects in the scene. The 322 participants experienced different combinations of video and audio in random order and were asked to evaluate each regarding its usefulness for real estate agents. They could not explore the scenes by changing their position, since movement in VR was controlled by the experimenter. Presence was measured by 14 questions including those used by Hendrix and Barfield (1996). The Presence-Ratings obtained on a 1–100 scale were quite high compared with other studies and can be found in Table 1 they show a statistically significant effect of adding sound to the VR.

Larsson et al. (2007) visually presented a virtual representation of a church in Gothenburg (Sweden) using LCD shutter glasses and a VR-CUBE with 3D graphics displays on all four walls and the floor. The virtual sound was organ music, reproduced via eight active loudspeakers in each corner of the VR cube to achieve surround sound. The 30 participants (26 male, 4 female) had the task to find five numbered cubes and navigate through them in correct order. For measuring presence, the authors used the short version of the Swedish viewer-user presence Questionnaire (SVUP; Larsson et al., 2001) with 18 items. The results in Table 1 show that the presence ratings and effect sizes coincidentally are almost the same size as the ones obtained by Hendrix and Barfield (1996), but the standard deviations are much smaller. Like in most other studies, reproducing sound significantly increased the ratings of presence.

The methodological diversity of the studies reviewed and their somewhat inconsistent results make it difficult to draw any further conclusions beyond stating that it is likely that sound enhances perceived presence in VR somehow. Therefore, we decided to further investigate the issue using a modern, stereoscopic head-mounted VR display and stereophonic sound drawing on two sources: (a) environmental sounds, and (b) the self-generated sound of footsteps produced by walking in the VR.

One of the first decisions to be made was how to design the sounds so they would lead to findings adding to the already existing body of research. We wanted to benefit from the “temporal nature of sound” implying that “something is happening” (Larsson et al., 2010), so the variation of sound over time should be noticeable. Furthermore, referring to Ramsdell's (1978) three levels of sound, all of the previous studies had used sound on a “primitive” level (as opposed to the “social” and “signal” levels), with the goal of comfortably blending the participants into the virtual reality around them. We decided to remain consistent with that approach, thereby avoiding the need to act in response to auditory signals.

The term “soundscape” describes an acoustic environment as perceived or experienced and understood by a person (International Organization for Standardization, 2014). According to Serafin and Serafin (2004), a soundscape for a virtual reality may be made up of (a) the background noise used to create a general atmosphere (e.g., wind, traffic sounds, music) and (b) predictable and impulse-driven sound-events (e.g., steps, doors opening). For the present study, we decided to use a diffuse nature soundscape with wind and birds for the former, and the sound of the participant's own footsteps for the latter. There are just a few studies dedicated to investigating the sound of footsteps in VR:

In a study by Nordahl (2005), 20 participants were asked to wear a HMD, headphones and sandals equipped with pressure sensors while moving through a VR rendering the interior of the Prague Technical Museum. They were randomly assigned to a visual or an audio-visual condition, the former merely presenting a 3D-reconstruction of the museum, the latter additionally including self-generated step sounds triggered by the pressure-sensitive sandals and delivered via headphones. The results showed that the sense of presence—measured by the SVUP—was significantly higher in the bimodal (*M* = 5.35, *SD* = 0.39) than in the unimodal condition (*M* = 4.64, *SD* = 0.63). Since this suggests that reproducing one's own step-sounds should increase presence in virtual realities, we chose step sounds as the “impulse-driven” sound events.

Recognizing the participants' actual steps and playing back appropriate sounds synchronously poses technical challenges. Visell et al. (2009) suggest using either instrumented floors, a rigid surface equipped with sensors and actuators (e.g., Pinkston, 1994; Paradiso et al., 1997; Cook, 2002; Law et al., 2008; Nordahl et al., 2011) on which the users can walk wearing ordinary shoes, or instrumented shoes with sensors integrated in the sole or insole. The sensors can trigger the playback of footstep sounds by means of wearable 3D (binaural) auditory display or by wearable loudspeakers to permit proper spatial auralization (e.g., Paradiso et al., 1999; Benbasat et al., 2003; Nordahl, 2005), or with actuators integrated into the sole or insole to permit additional vibrotactile stimulation (e.g., Nordahl et al., 2010). In some studies, the playback of footsteps has even been adapted to reflect the walker's weight (Tajadura-Jimenez et al., 2015) or properties of the surface stepped upon (Serafin et al., 2010). For the present study, in order to be independent from instrumented floors or shoes, our goal was to compute the probability and moment a step is executed from sensor data of the HMD.

Similar efforts had been made by Slater et al. (1995): they trained a neural network for 10 min to recognize steps based on personal movement patterns of the HMD tracker position values of participants walking in place while being presented an indoor virtual environment, achieving a step recognition rate of 91%. By contrast, the algorithm independently developed in our laboratory by Caserman et al. (2016) not only estimated the probability of a step being executed but also the probability of that being taken with the left or the right foot, in order to reproduce corresponding step sounds in time.

For the present investigation, the principal research question was which sounds would enhance presence most. Would it be enough to just cancel the noises from the lab, particularly the treadmill-sounds, i.e., to isolate the user from the environment in order to increase presence in the VR? Would it be helpful, if sounds triggered by the user's footsteps on the treadmill were presented in addition to noise canceling? Would a soundscape which corresponded to and enhanced the virtual environment have a greater effect? Or would the combination of virtual steps and soundscape produce the highest degree of presence?

To answer these questions we investigated participants' sense of presence while moving through a VR by walking on a treadmill. Its audio component was varied in a within-subjects design by which the presentation of footstep sounds triggered by the participant and of an environmental soundscape were factorially combined in an on/off fashion and—in Experiment 1—supplemented with a no-headphones control condition.

## Experiment 1

### Method

#### Participants

Thirty-six participants (11 male, 25 female) took part in Experiment 1, mostly psychology students with ages ranging from 18 to 47 years (M = 25.5, SD = 6.14). All participants claimed not to have any impairment of hearing or movement. Visual impairment was tolerated up to one diopter, larger refractive errors only, if corrected by glasses or contact lenses. Due to the technical requirements of the treadmill, the participants were allowed a maximum height of 185 cm, as not to touch the front of the treadmill while holding onto the handle, and a maximum weight of 150 kg. This study was carried out in accordance with the recommendations of the Ethics Code of the American Psychological Association. The protocol was approved by the central ethics commission of TU Darmstadt. All subjects gave written informed consent.

#### Study Design

Experiment 1 was conducted as a one-factorial, within-subjects design with five levels. The type of background sound was varied (see Table 2) to assess its influence on the dependent variable, the feeling of presence in the virtual reality.

**Table 2:** The sound conditions evaluated in Experiment 1.

**Condition**	**Noise canceling headphones?**	**Sounds presented in VR?**	**Sounds from the laboratory**
No headphones	NO	NO	Clearly audible
Noise-canceling	YES	NO	muffled
Steps	YES	YES (virtual steps)	muffled
Soundscape	YES	YES (Soundscape)	muffled
Steps & Soundscape	YES	YES (virtual steps, Soundscape)	muffled

#### Experimental Setup

Experiment 1 was conducted in a robotics lab with other people being present and at times talking in low voices. During each trial, the participants wore an Oculus Rift DK2 HMD allowing for stereoscopical presentation of visual stimuli on two OLED displays with a resolution of 960 × 1,080 pixels per eye and a refresh rate of up to 75 Hz (XinReality, 2016), as well as Bose QuietComfort 25 Acoustic noise-canceling over-ear headphones. To overcome the problem of walking about within the limited space of the laboratory, the participants actually walked on a fitness-training treadmill (Altmark-Trading, model GV5052W). The Virtual Reality and the sounds were reproduced in Unity (Version 5.4.0b13).

#### Visual Virtual Reality

**Figure 1:**
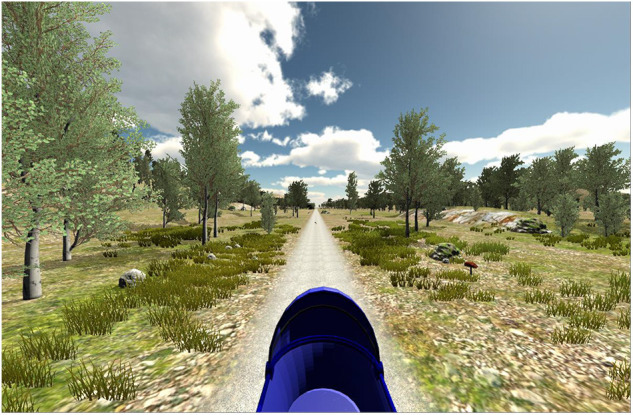
The virtual environment employed in both experiments: a nature walk along a gravel path leading straight through a hilly countryside.

The virtual reality simulation was implemented in Unity 5. It provided the viewer with a nature walk along a gravel path leading straight through hilly countryside (see Figure 1), passing trees, rocks, bushes, mushrooms and benches, as well as several barns with fences, a small village with a church and power pylons. In the course of the walk, at times butterflies fluttered around. The weather was sunny, although there were some clouds in the sky. The participant moved in first-person perspective with an avatar pushing a stroller (thereby copying the actual body-posture of holding on to the treadmill-handle).

#### Audio Virtual Reality

Our soundscape consisted of sounds with a localizable origin, e.g., the ringing of church bells^1^ originating from the bell tower or the sound of the steps—if presented—triggered by the participant's movements. In addition there were nature sounds without localizable origin^2^, namely the ambient nature soundscape, which included, among other things, birds twittering, ravens calling, a deer barking and the very faint bubbling of a nearby mountain stream. The sounds were delivered using the built-in Unity 5.1 audio rendering system. That is, localizable sounds (e.g., church bells) were assigned to the respective objects in the scene using the default settings, and the listener was assumed to be at the avatar's head position (or camera position). Thus, the sounds were spatialized by the default mechanisms provided by Unity, i.e., adjusting the gains of the left and right ear channels based on the angle between the listener and the source, and attenuating the volume (in decibels) in a linear fashion as a function of distance. “This provides simple directional cues for the player on the horizontal plane” (Unity Technologies, 2019). The sounds without localizable origin, particularly the ambient nature soundscape, were not assigned to any particular location.

The footstep sounds were recordings of actual steps on a gravel surface, downloaded from the Internet^3^. To enable sound reproduction synchronous with the user's actual steps, the recording, a sequence of steps, was cut so that single footsteps on a gravel surface could be reproduced when required. To avoid an artificial impression produced by reoccurring sequences, for the left foot as well as the right foot, eight different recordings were made available from which an algorithm randomly chose one to be reproduced, not matching the one played right before. The footstep sounds were positioned in space by assigning them (in Unity) to the right and left foot of the avatar, respectively.

All sounds were provided in stereophonic WAVE format, subsequently compressed to the Vorbis format by the Unity software, and played back preserving the sampling rate of 44.1 kHz. The sound of the church bells (37.4 s) and the soundscape (7 min) were played when called for and then looped, the footstep-sounds (between 0.626 and 0.783 s) were only reproduced when triggered.

For timing the reproduction of the step-sounds, Caserman et al. (2016) developed an algorithm estimating the probability of the walker executing a step from the Oculus rift orientation data, which was used and evaluated in our experiments. They used four different step detection algorithms using linear HMD data about acceleration, linear and rotational velocity, as well as linear position measurements. The accuracy of (a) a low-pass filter and (b) an acceleration-based step-detector ranged between detection rates of about 50% at worst and more than 90% at best. A (c) velocity-based step detector showed rather mixed results depending on walking speed. A (d) position-based step detector provided the best results at all walking speeds, detecting more than 90% of the steps in most trial runs. Furthermore, an adaptive threshold for peak searching and an adaptive side decision mechanism estimated if a step had been taken and on which side it had been executed. The values received by the four different algorithms were weighted as follows: acceleration with 10%, velocity with 10%, position with 70%, and the low-pass filter with 10%. The weightings are subsequently summarized by two variables “voteLeft” and “voteRight,” which together sum up to 100%. If one of the variables surpasses 70% (which practically means, that position plus at least one other detector indicate a specific side), that side is set as being the origin of the actual step. If it does not match the last recognized step, which means, if a change happened, the step is counted as a new step and the process begins again. Unless 70% were exceeded, no step was counted and therefore no step sound was replayed which at times led to gaps in step reproduction. Although in objective evaluations of the algorithm at walking speed (Caserman et al., 2016) the step detection rate was rather high (between 92 and 100%), it still remains to be shown what the user perception might be, which was assessed in the present study.

#### Presence Questionnaire

For Experiment 1, items measuring presence were eclectically chosen from published questionnaires. Two questions assessing presence and realism were 1-item-scales taken from Hendrix and Barfield (1996). In addition, some items which might suggest design implications for immersive VR sounds were taken from the Witmer and Singer (1998) presence questionnaire, which had been translated and shortened (questions referring to actions, interactions, or manipulation of the virtual environment, to haptics and interaction devices were not included since they did not apply to the present study). The questionnaire was then extended by some questions specifically focusing on the effect of the audio components. Five different versions of the questionnaire were created, in which the questions were randomized to reduce memory effects. The resulting questionnaires were always presented in randomized order.

#### Procedure

In each experimental condition, the participant walked on the treadmill with a velocity of 2.5 km/h while seeing a virtual reality presented by an Oculus Rift HMD and wearing headphones (except in the “No Headphones”-condition). The duration of exposure to the virtual reality for a given condition was varied between 3:40 and 5:00 min to permit varying estimations of elapsed time as a dependent variable assessing a potential loss of the participant's sense of time. During the exposure, the participant was asked to count fly agarics aiming to capture the participant's attention. After each exposure to a given VR/audio condition, the participants filled out the presence questionnaire before entering the next experimental condition. The order of the experimental conditions and the duration of the trials were independently randomized by using multiple 5 × 5 Latin squares.

## Results

### Presence and Realism

**Figure 2:**
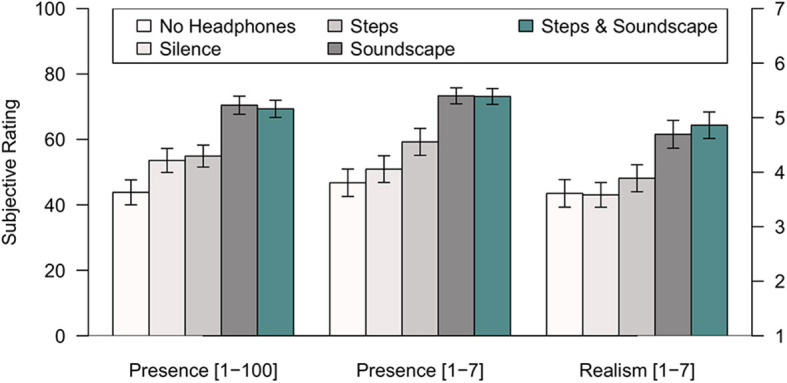
Ratings of presence and realism in Experiment 1. Mean single-item ratings based on the questions proposed by Hendrix and Barfield (1996) are displayed along with standard errors of the means for the five experimental conditions identified in the legend.

The subjectively perceived sense of presence had been assessed by two questions from Hendrix and Barfield (1996), the first comparing the VR to reality on a scale from 1 to 100 (1: “no presence at all,” 100: “presence like in the real world”), the second assessing the feeling of “being there” on a scale from 1 to 7 (1: not at all, 7: very much). The results (see Figure 2) show that the sense of presence increases from a minimum in the “No Headphones”-condition, over the “Noise-Canceling”-, “Steps”- condition up to the maximum reached in the “Soundscape”- and “Steps & Soundscape”- conditions.

Realism had been assessed by one question from Hendrix and Barfield (1996) on how realistic the virtual environment appeared on a scale from 1 to 7 (1: not at all, 7: very much). Figure 2 shows mean responses ranging from 3.58 to 4.83: While the conditions “No Headphones,” “Noise-Canceling,” and “Steps” resulted in similar ratings of realism, the conditions involving soundscapes were judged to be considerably more realistic.

Welch two-sided *t*-tests were conducted to test whether the presence and realism-ratings in the condition without headphones significantly differed from those in the no-sound, noise-canceling condition, but no significant effects were found, suggesting that presence did not depend on whether the participant wore headphones or not.

In order to assess the auditory effects of playing back step-sounds or adding an ambient “Soundscape,” two-way analyses of variance (excluding the “no Headphones” condition) were conducted, thereby analyzing the effects of including “Steps” (played back or not) or an ambient soundscape (on or off). For the presence-ratings from 1 to 100, the factor “Soundscape” was highly significant, *F*_(1, 35)_ = 48.95, *p* < 0.001, with a large effect size (η^2^ = 0.583), while the factor “Steps” had no significant effect, *F*_(1, 35)_ = 0.003, *p* = 0.96, nor did their interaction, *F*_(1, 35)_ = 0.531, *p* = 0.471. The results of the presence-ratings from 1 to 7 paint a similar picture. With respect to realism, the factor “Soundscape” also resulted in highly significant differences, *F*_(1, 35)_ = 42.48, *p* < 0.001, with a large effect size (η^2^= 0.548), while the factor “Steps” had no significant effect, *F*_(1, 35)_ = 1.52, *p* = 0.226, nor had their interaction, *F*_(1, 35)_ = 0.173, *p* = 0.680. These results imply that Presence and realism are increased by playing back an appropriate soundscape, but not by adding self-triggered sounds of footsteps.

### Involvement, Realism, and Distraction

**Figure 3:**
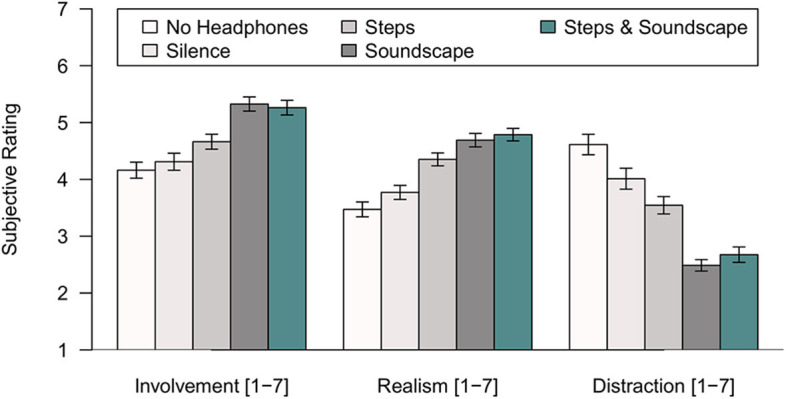
Means and standard errors for the multi-item scales of involvement, realism, and distraction (Witmer and Singer, 1998) as a function of the sound condition in Experiment 1. Each mean is based on aggregation across 5–7 items per scale and 36 participants.

The Witmer and Singer (1998) involvement-, realism-, and distraction scales were evaluated by computing means across 5–7 items. As Figure 3 shows, there were no differences between the two control conditions (No Headphones, Noise Canceling), except that distraction was judged to be greater without headphones, *t*(69.96) = −2.342, *p* < 0.05. Playback of a soundscape increased both involvement and realism, and simultaneously decreased distraction by the lab environment (ANOVAs, all *p* < 0.001, effect sizes ranging between η^2^ = 0.562 and η^2^ = 0.719). Including the sound of the participants' footsteps in the VR produced an effect of its own on the realism scale only, *F*_(1, 35)_ = 10.409, *p* < 0.001; η^2^ = 0.229. The significant interaction observed between the presence of a soundscape and the playback of steps on the realism scale, *F*_(1, 35)_ = 7.235, *p* < 0.05, η^2^ = 0.171, confirms the impression that providing a soundscape and adding step sounds does not increase presence in an additive fashion.

### Visual Aspects and Auditory Aspects

For the Witmer and Singer (1998) visual aspects scale, the mean of three items assessing the ability to visually search the VR, the visual quality and visual involvement was calculated. As Table 3 shows, neither the group means nor the standard deviations differ much between the conditions. A two-way analysis of variance assessing the influence of the factors “Soundscape” and “Steps” on the Witmer & Singer visual aspects scale turned out insignificant. That suggests that perception of the visual appearance of the VR was not influenced by variations of the audio component.

**Table 3:** Descriptive statistics on the Witmer and Singer (1998) visual and auditory aspects scales for Experiment 1.

	**Visual aspects** **M(SD)**	**Auditory aspects** **M(SD)**
No Headphones	4.556 (0.973)	NA
Noise-canceling	4.519 (0.951)	NA
Steps	4.713 (0.789)	4.337 (0.967)
Soundscape	4.685 (0.891)	5.582 (0.733)
Steps & Soundscape	4.713 (0.933)	5.472 (0.605)

*For the two conditions without sound, no values were calculated, since the audio questions had not been included*.

For the Witmer and Singer (1998) auditory aspects scale, a mean was calculated across five items assessing the ability to identify and localize sound, as well as audio quality, auditory involvement and how much real sounds from the laboratory disturbed the impression (polarity reversed). Table 3 shows the results for the auditory aspects scale, the condition with steps only resulting in a mean of 4.34 in contrast to ~5.5 in the two conditions with Soundscapes, suggesting that it is only between the Soundscape and the non-Soundscape conditions that an audio effect occurs. A one-factorial analysis of variance turned out significant, *F*_(2, 70)_ = 33.9, *p* < 0.001, with a large effect size (η^2^ = 0.478). Pairwise comparisons using paired *t*-tests with Bonferroni corrections clarified that there are significant differences between the Steps condition and the two conditions involving a soundscape, the latter ones being rated significantly higher in audio quality. In conclusion, the differences are due to adding the soundscape whereas adding step sounds did not result in significant effects.

### Synchronicity of the Steps

To evaluate the step reproduction algorithm, participants also assessed whether they felt their actual steps and the steps they heard in the virtual reality to be synchronous by making a rating on a seven-point scale labeled at the extremes from “not at all” (synchronous) to “very” (synchronous). Coding the response categories as 0–6, in the “Steps” condition, the average synchrony rating was *M* = 2.33 (*SD* = 1.57), in the “Steps & Soundscape” condition it was *M* = 2.81 (*SD* = 1.56), thus in both cases only slightly below the midpoint of the response scale. To evaluate, whether the shortcomings in the playback of step sounds contributed to reducing presence, correlations between the synchronicity ratings and various measures of presence and its sub-dimensions were computed. The strongest correlation was observed between synchronicity and the Hendrix and Barfield (1996) single-item realism scale, *r* = 0.34, *t*(70) = 3.03, *p* < 0.01, suggesting that the more the step sounds were perceived as synchronous with one's own gait, the more realistic the environment was perceived. Slightly weaker correlations were obtained with involvement in the VR, *r* = 0.25, *t*(70) = 2.19, *p* = 0.03, and with distraction by extraneous events *r* = −0.24, *t*(70) = −2.0814, *p* = 0.04.

### Losing Track of Time

Many studies assess whether the participant has the feeling of losing track of time, so we asked the participants to estimate the time they had spent in the VR in a given condition and calculated the difference to the actual time elapsed. In general, the results show that there was a tendency to underestimate the time spent in the virtual reality. The means differed considerably by condition, but so did their standard deviations, thus failing to produce significant differences in time estimation accuracy between conditions. Interestingly, however, a small but significant Pearson product-moment correlation between the Witmer and Singer (1998) question whether participants had the feeling of losing track of time and their actual time estimates was found, *r* = 0.224, *t*(178) = 3.068, *p* < 0.01, suggesting that the more participants felt they lost track of time, the longer they estimated their time spent in the VR.

### Perceived Sounds

Two questions assessed if the participants heard sounds from the laboratory environment or the virtual environment, respectively. Participants reported hearing the noise of the treadmill, their own steps, technical sounds (e.g., the computer or a small electric motor), or human-produced sounds (e.g., opening and closing doors, conversations in low voices in the laboratory or on the hallway). While there were only few hearing nothing at all in the conditions “No Headphones” and “Noise-Canceling,” that number increased to 12 participants in the “Steps” condition, 20 in the “Soundscape” condition and 18 in the “Steps and Soundscape” condition. That suggests that reproducing sounds like steps or a soundscape in itself serves to block out auditory distractors from the actual surrounding.

With respect to hearing sounds in the virtual environment, the perception was quite rich, including wind noises, wings flapping, distant thunder, the humming of flies and insects and the chirping of crickets. One participant even reported hearing a chainsaw, although there was no such sound in the soundscape.

## Discussion

Experiment 1 demonstrates that the reproduction of an appropriate soundscape along with the visual VR has highly significant effects on Presence, Realism, Involvement, Distraction and (naturally) the “Auditory aspects” of exposure to the virtual world, all with large effect sizes. Note, that the baseline condition without headphones produced an average presence rating (on a 1–100 scale) of 44 in comparison to a mean of about 70 for the conditions with soundscapes, thereby indicating that presence is considerably enhanced if the sensory information about the virtual environment is delivered in more than one modality. Of course, that also implies that the presence in the virtual reality is only about 70% of the presence which is perceived when interacting with a comparable real-world scenario.

Reproducing self-generated footstep sounds, however, neither produced significant effects on overall measures of presence, nor on its subscales. The only exception is the realism scale, suggesting that providing proprioceptive audio feedback may increase the authenticity of the virtual world.

In general, whether (noise-canceling) headphones are worn or not reduces distraction by the actual (laboratory) environment. That is consistent with responses to the open question on whether sounds were heard or not: reproducing sound like steps or a soundscape in itself helped to block out auditory distractors from the actual surrounding. Presence, involvement and realism, however, are not influenced by just wearing headphones. Thus, the active noise canceling only diminished distraction.

The time estimation task showed a general tendency to underestimate the time spent in the VR, but notably, although we offered spaces for entering minutes and seconds, nearly all participants rounded their estimates up or down to full or half minutes which is problematic because it diminishes resolution. That suggests looking for better ways to investigate the sense of losing track of time.

As to the authenticity of playing back user-triggered step sounds in the VR, the ratings of synchronicity of the steps were rather disappointing, ranging between 2 and 3 on the 0–6 scale, although it is evident that the reproduction of a soundscape enhanced the perceived synchronicity of the steps. Still, the algorithm calculating the steps should be improved in further studies.

## Limitations and Outlook

Although adding sounds to the visual virtual reality did greatly enhance presence for the conditions involving soundscapes, there was no significant increase in presence due to the presentation of step-sounds synchronized with the participant's gait on the treadmill. That might be due to the fact that the algorithm reproducing the steps is still suboptimal, generating gaps in the step sound presentation which are perceived to be asynchronous. Therefore, the step-reproduction algorithm was modified in order to re-assess the interaction of the availability of an ambient soundscape and the presentation of self-generated footsteps with an improved experimental setup.

Furthermore, the eclectic use of items and subscales from existing questionnaires may be problematic due to the unknown reliability and validity of the selection. Therefore, in Experiment 2, a full-length, psychometrically evaluated questionnaire was employed (IPQ, igroup.org, 1995–2016) to measure presence and its expression in various subscales.

## Experiment 2

### Method

#### Participants

Forty three participants took part in the study, three of whom had to be excluded due to technical difficulties during the presentation of the virtual world. Therefore, data analysis was conducted on 40 participants (11 males, 29 females) with ages ranging from 18 to 34 years (M = 22.95, SD = 4.44). The participants were all students, most of them of psychology. With respect to their usage of HMD's, 50% had never used one, another 45% had used an HMD between one and three times and only two participants had worn it 10 or 30 times, respectively. All participants signed an informed consent form explaining the study and informing them about their right to end participation at any time. The protocol was approved by the central ethics commission of TU Darmstadt. All subjects gave written informed consent.

#### Study Design

The second experiment was designed as a two-factorial, within-subjects design with two levels per factor (see Table 4). The two factors varied as independent variables were the playback of step sounds in VR (yes/no) and the presentation of an appropriate soundscape (yes/no). The dependent variable was presence, the feeling of being there as measured by the IPQ Presence Questionnaire (igroup.org, 1995–2016). The order of the experimental conditions was randomized by using multiple 4 × 4 Latin squares. The no-headphones condition of Experiment 1 was omitted.

**Table 4:** The different conditions evaluated in Experiment 2.

**Condition**		**Soundscape reproduced**	
		**No**	**Yes**
Virtual steps reproduced	No	Noise-canceling	Soundscape
	Yes	Steps	Steps & Soundscape

#### Experimental Setup and Procedure

The procedure, the Virtual Reality and the sounds used (Steps and Soundscape) were the same as in Experiment 1. The experimental setup was changed insofar as the second experiment was conducted in a windowless laboratory room with hardly any background noise, except for the constant hiss of the air condition, which was almost completely suppressed by the noise-canceling headphones.

Furthermore, small changes were made to the step-sound algorithm as to enhance user perception. The position was now weighted 100% which means, the data from the position detector alone were used to estimate the execution of a step. The other detectors (acceleration, velocity, and low-pass filter) were no longer taken into account. Furthermore, a step was always triggered when the momentary duration of a step exceeded the medium duration of previous steps in order to avoid gaps in step reproduction.

#### Presence Questionnaire

The focus of Experiment 2 was to measure changes in presence and realism using an established full-length presence questionnaire. We chose to use the IPQ (i-group Presence Questionnaire; igroup.org, 1995–2016), because it is relatively short and a German version is available which has been previously validated (Schubert et al., 2001). The IPQ measures presence with a single-item-scale assessing the overall impression of having been in the computer-generated world. Furthermore, it has three multi-item scales for different aspects of presence, namely realism (the feeling that the virtual world seemed real), spatial presence (the feeling that one is really located and acting in the virtual world and that it is more than just pictures), and involvement (the feeling of no longer being conscious of the real world). In addition, a question assessing presence on a 1-item-scale from Hendrix and Barfield (1996) was included, to facilitate comparisons with Experiment 1. The questionnaire was then extended by questions assessing further auditory aspects, time estimation, and two open questions assessing which sounds had been heard in the virtual and the laboratory environment, respectively. Five different versions of the questionnaire were created, in which the questions were randomized to reduce memory effects. These questionnaires were always presented in randomized order.

## Results

### Presence

**Figure 4:**
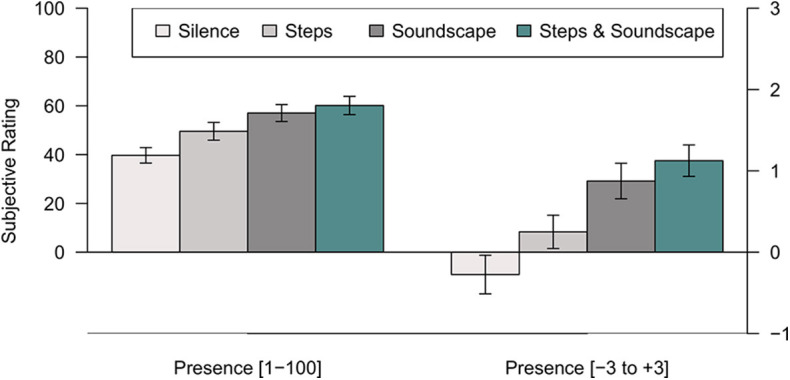
Means and standard errors for the Hendrix and Barfield (1996) presence scale (left; range: 0–100) and the IPQ G1 single-item presence scale (right; range: −3 to +3) as a function of the sound condition in Experiment 2. Each data point is based on ratings made by 40 participants.

Responses to the IPQ single-item question show that presence, the impression of “being there” in the computer-generated environment, rated on a scale from −3 to +3 increases from a minimum in the “Noise-canceling” control condition, over the “Steps” condition up to the maximum reached in the “Soundscape” and “Steps & Soundscape” conditions (see Figure 4). A two-factor, repeated-measures analysis of variance with the factors Steps and Soundscape showed significant main effects of the factor Steps, *F*_(1, 39)_ = 5.821, *p* = 0.021, η^2^= 0.130, and the factor Soundscape, respectively, *F*_(1, 39)_ = 24.512, *p* < 0.001, η^2^ = 0.386. The interaction of both factors was not statistically significant, *F*_(1, 39)_ = 0.831, *p* = 0.368.

The single-item question from Hendrix and Barfield (1996) (1: “no presence at all,” 100: “presence like in the real world”), paints a similar picture: the mean ratings are rank-ordered in the same fashion (see Figure 4). A two-factor analysis of variance on the presence-ratings from 1 to 100 revealed a significant main effect of steps, *F*_(1, 39)_ = 11.797, *p* = 0.001, η^2^ = 0.232, as well as of soundscape, *F*_(1, 39)_ = 41.518, *p* < 0.001, η^2^ = 0.516. The interaction of steps and soundscape, *F*_(1, 39)_ = 5.361, *p* = 0.026, η^2^ = 0.121, was also statistically significant, suggesting that the introduction of step sounds had a greater effect on presence when it was the only auditory enrichment provided, as compared to when it was combined with an ambient soundscape.

### Realism, Spatial Presence, and Involvement

The mean of four items each was calculated for the IPQ-subscales “Realism,” “Spatial Presence,” and “Involvement.”

With respect to the Realism of the VR, the ranking is the same as for the presence scales (see Table 5): the lowest value is reported for the Noise-canceling condition, followed by the Steps-, the Soundscape- and the Steps & Soundscape condition which differ from each other by about 0.3 scale units (see Table 5). A two-factor analysis of variance on the factors Steps and Soundscape showed significant main effects of the factor Steps, *F*_(1, 39)_ = 12.939, *p* < 0.001, η^2^ = 0.249, and the factor Soundscape, respectively, *F*_(1, 39)_ = 20.185, *p* < 0.001, η^2^ = 0.341. The interaction of both factors was not statistically significant, *F*_(1, 39)_ = 0.854, *p* = 0.361.

**Table 5:** Descriptive statistics on the IPQ scales Realism, Spatial Presence, and Involvement for Experiment 2: means and standard deviations for each experimental condition.

	**IPQ Realism [−3 to +3]**	**IPQ Spatial Presence [−3 to +3]**	**IPQ Involvement [−3 to +3]**
Noise-canceling	−1.375 (1.146)	0.17 (0.724)	−0.331 (0.827)
Steps	−0.838 (1.037)	0.16 (0.564)	−0.275 (0.814)
Soundscape	−0.613 (1.337)	0.285 (0.619)	0.231 (0.752)
Steps & Soundscape	−0.306 (1.184)	0.33 (0.478)	0.256 (0.746)

As Table 5 shows, as for Spatial Presence, all experimental conditions resulted in quite similar mean ratings and standard deviations. Consequently, none of the effects (of steps or soundscape) were statistically significant.

On the involvement scale the means for the conditions without soundscape are about the same, as are the means for the conditions providing a soundscape (see Table 5). Consistent with that observation, a two-factor analysis of variance on the factors Steps and Soundscape showed no significant main effect of the factor Steps, *F*_(1, 39)_ = 0.430, *p* = 0.516, while revealing a significant main effect for the factor Soundscape, *F*_(1, 39)_ = 36.983, *p* < 0.001, η^2^ = 0.487. The interaction of both factors was not statistically significant, *F*_(1, 39)_ = 0.026, *p* = 0.874.

### Visual Aspects and Auditory Aspects

As Table 6 shows, for the visual aspects scale the means and standard deviations do not differ much between conditions, the visual quality of the VR is judged to amount to ~4.5 on a 7-point scale, no matter what the audio condition; none of the subtle differences in means is statistically significant.

**Table 6:** Descriptive statistics on the Witmer and Singer (1998) visual and auditory aspects scale for Experiment 2.

	**Visual aspects** **(1–7)**	**Auditory aspects** **(1–7)**
Noise-canceling	4.581 (0.794)	NA
Steps	4.558 (0.862)	4.679 (0.869)
Soundscape	4.496 (1.068)	5.378 (0.643)
Steps & Soundscape	4.617 (0.974)	5.494 (0.695)

By contrast, on the auditory aspects scale the mean ratings tend to increase when a soundscape is added. A one-factorial analysis of variance shows that the auditory aspects ratings distinguish the three sound conditions, *F*_(2, 78)_ = 18.6, *p* < 0.001, η^2^ = 0.323. A pairwise *t*-test with Bonferroni correction clarifies that the difference is between the Steps-condition and the two conditions with Soundscape, the latter not differing significantly from each other. That suggests that replaying a soundscape affects auditory perception whereas replaying steps does not.

### Synchronicity of the Steps

As in Experiment 1, participants rated whether they felt their actual steps and the steps they heard in the VR to be synchronous. In the “Steps” condition, on average, the synchrony was rated as *M* = 3.225 (*SD* = 2.069) on the 7-point Likert scale, in the “Steps & Soundscape” condition as *M* = 3.675 (*SD* = 1.760), slightly above the midpoint of the scale and approximately half a response category higher than in Experiment 1. A Welch two-sample *t*-test confirmed that difference to be statistically significant, *t*_(148.7)_ = −3.103; *p* < 0.01. As in Experiment 1, a significant correlation between the rated synchronicity of the step sounds and the perceived realism of the VR was found, *r* = 0.40, *t*(78) = 3.89, *p* < 0.001, but in Experiment 2, synchronicity was also related to overall presence: A significant correlation was found with the 1–100 presence scale, *r* = 0.28, *t*(78) = 2.61, *p* < 0.05, and marginal significance when correlating synchrony ratings with the single-item presence scale of the IPQ.

## Discussion

In Experiment 2, the variation of essentially the same auditory features as in Experiment 1 (auditory playback of footstep sounds, introduction of a rich ambient soundscape) but with an improved step-reproduction algorithm produced highly significant perceptual effects of both features, where in Experiment 1 only the soundscape had been influential.

That was true for the sense of presence experienced in the VR as well as for its perceived realism, while the IPQ subscale of spatial presence was not affected, and the IPQ subscale of involvement only reflected the auditory enrichment by the soundscape.

The latter is somewhat surprising, since hearing one's own steps in the VR is intuitively highly likely to contribute to involvement. Note, however, that the step algorithm is still far from perfect as reflected in the low synchronicity ratings. That the spatial presence subscale was not sensitive to our manipulations of the auditory environment may be due to the fact that the sounds, though being somewhat localizable by the mechanisms provided in Unity (as with the sound of church bells waxing and waning as the listener passed by), were not specifically designed to provide directional audio cues, nor did the participants' task require the use of spatial auditory information. Rather, the soundscape used in the present research delivered diffuse, ambient information on sound sources (wind noise, birds twittering), much less than on their specific location.

## General Discussion

In two experiments, participants moved on a straight gravel path through a park-like virtual reality presented by a HMD while actually walking on a treadmill. During that experience the visual VR was supplemented with different types of audio exposure in order to study to what extent auditory stimulation enhances perceived presence.

An initial question for this research was, whether pure noise-canceling without reproduction of sound would lead to mild feelings of isolation or “deadness” as reported by Ramsdell's (1978). As shown in Experiment 1, that did not seem to be the case: Presence, involvement, the quality of the visual experience, and the realism of VR exposure were unchanged, no matter whether participants wore noise-canceling headphones or no headphones at all. On the contrary, distraction by the actual environment was reduced when wearing (silent) noise-canceling headphones, and even more so, when these headphones played back the participants' own steps, or an appropriate soundscape.

The variation of the sounds presented via headphones to complement the VR had a strong influence on perceived presence, realism and the auditory aspects scale, so presenting a plausible soundscape or self-triggered step sounds evidently made participants feel more present and the virtual reality appear more real. Notably, the effect of including the soundscape was generally larger than the effect of adding step sounds which, for some measures, even turned out to be insignificant in Experiment 1.

As regards retrospective ratings of presence, in conditions with the visual VR only, and without sound, people experienced between 40 (Experiment 2) and 54 (Experiment 1) percent of real-world presence in our virtual “walk-in-the-park” scenario. By contrast, in both experiments, presence ratings increased by some 15–20 percentage points when both step sounds and the ambient soundscape were added. In general, ratings on all quality-related scales in the Steps & Soundscape condition were a little lower in the second experiment compared to the first which might indicate that the second sample was more skeptical, though that is not supported by discrepancies in their previous exposure to VR, for example.

Notably, spatial presence, the feeling that one is really located and acting in the virtual world and that it is more than just watching a movie, was not influenced by the sounds at all in Experiment 2. Further research might investigate whether achieving high spatial presence is simply difficult, or whether our failure to provide it is due to the particular sounds chosen.

The perceived synchronicity of the steps was disappointingly low in both experiments, though a simplification of the step-reproduction algorithm slightly improved synchronicity ratings in Experiment 2. Interestingly, in both experiments, the perceived synchrony of the steps appeared to have an effect on participants' sense of presence, particularly as measured by the “realism” subscales. It is conceivable that the mechanism by which ill-fitted step sounds reduce presence is the participant's sense of agency, the perception of “being in action or of exerting power” (Nowak and Biocca, 2003, p. 483), in this case, the perception of controlling the movements, which in turn might have lowered the feeling of presence.

For integrating our study into the already existing body of research, we compared our results regarding presence and realism to those of earlier studies: mean scale values and effects sizes (comparing sound vs. no-sound conditions as in Table 1) are generally quite similar, despite huge differences with respect to the kinds of visual and auditory VR used. Note that the “ceiling” for presence and realism measured on 100-point scales in a VR laboratory setting appears to be near 70%; that is true both for the older studies (Table 1), as for the present one (Figures 2, 4). Generally, however, it may quite safely be concluded that presence is enhanced by the reproduction of sound. Presence ratings from 0-100 tend to average about *M* = 45–50% in conditions with no sound and about *M* = 60–70% in conditions with sound, with standard deviations of about 20. In terms of (statistical) effect sizes, there is not much published evidence yet, but in the present study, reproducing a soundscape influenced different aspects of presence with medium to large effect sizes ranging from about η^2^ = 0.40 to 0.72, whereas reproducing step-sounds alone resulted in effect sizes ranging from about η^2^ = 0.13–0.25. It should be noted, though, that due to the imperfections of the step reproduction procedure, the effect of adding self-triggered step sounds may be underestimated by the present research.

## General Limitations and Outlook

Clearly, the present study has a few inherent limitations which should be made explicit. In our view, they primarily concern (1) the research design employed, (2) the method of measuring presence, and (3) the implementation of audio enrichment chosen.

In comparing the different audio conditions, we chose to use a within-subjects design, where each participant experiences and rates each of the 4–5 audio conditions. That will undoubtedly reduce error variance due to inter-individual differences in scale usage, it is likely to enhance contrast between conditions (by direct, successive comparisons), but on the downside exposure to all audio conditions will increase the transparence as to what is being investigated, entailing the risk of encouraging participants to comply with the presumed research goals. That the results were replicated with two different samples, may serve to somewhat weaken that potential criticism. Nevertheless, replicating the present study using a between-subjects design and/or objective measurements might be called for.

As to the latter point, note that for all dependent measures, we used questionnaires to be completed retrospectively, after the actual experience. Therefore, less subjective and more continuous measurement methods are highly desirable, for example continuous measurement of presence while the participant is exposed to the virtual world. A better way to measure the phenomenon of losing track of time might also be of interest, as are objective/psychophysiological indices of presence (e.g., Witmer and Singer, 1998; IJsselsteijn et al., 2000; Slater et al., 2009).

Clearly, the algorithm used for step reproduction could be further improved, since the participants' perception of the synchronicity of auditory footstep playback varied a lot and was judged to be mediocre on average. Rather than further enhancing the HMD-sensor-based algorithm, one might also explore different methods for estimating the occurrence of steps, such as instrumented shoes or floors.

Finally, it should be noted that the present study is somewhat constrained by the choice of sounds used to supplement the visual VR, and by the technology employed to deliver them. The two types of sounds used in the experimental conditions implemented were: (a) Sensor-triggered, extraneous footstep sounds synchronized to the actual gait of the participant, and (b) a diffuse, globally enveloping nature soundscape providing a background “sound texture” rather than discrete auditory events. The null effect of our audio manipulations on the “spatial presence” scale and the rather small effects on “involvement” (Experiment 2) still have to be explained. Future studies might explore sounds that are more useful for orientation in the VR, or even instrumental in solving a spatial task, potentially including the “signal” or even “social” levels of usage of hearing postulated by Ramsdell (1978) over and above the “primitive” level the present study focuses on.

Likewise, in the present study, the placement of sounds in the environment was accomplished employing the basic methods supplied by the Unity software used to construct the VR. Future research might explore more sophisticated methods of rendering audio in VR, such as wave field synthesis (Ahrens et al., 2014), ambisonics (Shivappa et al., 2016), or binaural synthesis via head-related transfer functions (HRTFs; Hammershøi and Møller, 2005; Rummukainen et al., 2018; Ahrens et al., 2019) in order to maximize the potential of the auditory channel for contributing to a sense of presence in multimodal virtual realities.

## Data Availability Statement

The datasets generated for this study are available on request to the corresponding author.

## Ethics Statement

The present study was reviewed and approved by central ethics commission of Darmstadt University of Technology, Darmstadt, Germany (EK 19–2015). The patients/participants provided their written informed consent to participate in this study.

## Author Contributions

AK and WE contributed jointly to conception and design of the study, contributed to the statistical analysis and contributed to manuscript revisions, and read and approved the submitted version. AK performed the literature search, set up the visual and auditory VR, and collected the data and wrote the first draft of the manuscript. WE revised and complemented the manuscript.

### Conflict of Interest

The authors declare that the research was conducted in the absence of any commercial or financial relationships that could be construed as a potential conflict of interest.

## Footnotes

^1^http://www.soundsnap.com/node/35104

^2^https://soundcloud.com/jacek-maslanka/beskid-mountains-ujsolyregulus- blackcap-raven-and-roe-deer-near-mountain-stream

^3^http://www.soundsnap.com/node/49918
